# Supplementary material for: Deep neural networks integrating genomics and histopathological images for predicting stages and survival time-to-event in colon cancer
Source: PLoS One. 2024 Sep 3;19(9):e0305268. doi: 10.1371/journal.pone.0305268 (PMC11371203; doi:10.1371/journal.pone.0305268)
Supplement: Attachment — Submitted filename: Review.docx [file pone.0305268.s001.docx]

This article has established an integrated deep neural network for the staging classification of colon cancer,stratifying samples into low or high-risk survival groups.There is unexplained diverse variation within the predefined stages of colon cancer. This variation can be observed when utilizing only features from either genomics or histopathological whole slide images as prognostic factors. The unraveling of this variation is expected to lead to enhanced staging and improved treatment outcomes.

Here are my suggestions:

1. This article has a certain degree of novelty.The novelty of colon cancer group can be made by the data of mRNA, miRNA and DNA methylation make,but if the practical application of this group is further elaborated, this paper will be more perfect.
2. If there is more clear introduction on how the randomness of the data is assured,the article will be better.
3. The model established in this article was applied data from open databases without external data validation. It is desirable that more data are used to verify the accuracy of the model in further research,the application scope of this model will be wider.
