# Supplementary material for: Deep neural networks integrating genomics and histopathological images for predicting stages and survival time-to-event in colon cancer
Source: PLoS One. 2024 Sep 3;19(9):e0305268. doi: 10.1371/journal.pone.0305268 (PMC11371203; doi:10.1371/journal.pone.0305268)
Supplement: Attachment — Submitted filename: Response to Reviewer comments PONE-D-23-30061.docx [file pone.0305268.s002.docx]

Dear Editor,

We thank the reviewers for the constructive and thoughtful comments and suggestions on our manuscript originally entitled “Ensemble Deep Neural Networks (EDCNN) integrating genomics and histopathological images for predicting stages and survival time-to-event in colon cancer” and amended to “Deep Neural Network (DNN) integrating genomics and histopathological images for predicting stages and survival time-to-event in colon cancer” upon a comment from one of the reviewers (Manuscript Number: PONE-D-23-30061). We have revised the manuscript to address each of the comments. Below are our point-to-point responses to the reviewers’ comments.

The modifications in the manuscript are highlighted within the “Revised-PONE-D-23-30061-with-track.docx” document. We also provided an unmarked version of the revised document (Revised-PONE-D-23- 30061-unmarked.docx) upon your request.

Sincerely,

Dr Zeyneb Kurt,

Lecturer in Data Science,

The University of Sheffield, The Information School

**Reviewer #1:**

**Comment1:**

Thank you very much for sharing this interesting work that could add to the body of existing works on the study of multimodal data for prediction of patient diagnosis, and outcomes. The authors applied AI methods to investigate the use of three types of genomic data, and histopathology images for predicting cancer stage, and survival risk in colon cancer patients. Introduction:
“According to the World Health Organization 2020 report, cancer is the leading cause of death accounting for over ten million deaths”. Please provide a citation for this statement.

**Response 1:**

Thank you to the reviewer for their valuable comment. Relevant references have been added as recommended for our statement highlighted in the reviewer’s comment (page 2, lines 28 and 36 in the unmarked document),

**Comment2:**
“The complex features comprising the tumor size, the extent at which the cancer has spread to nearby lymph node, and whether the cancer has metastasized, which is the extent the cancer has spread to other parts of the body from the primary tumor still require the development of an efficient and effective model for accurate and improved classification and effective treatment management.” Please provide more clarity to this statement to make it obvious to a wider audience that the features mentioned are components of TNM staging. Since staging is the focus of this work.

**Response 2:**

Thank you to the reviewer for their comment. Upon reviewer’s comment, we have re-edited that portion of the paper and made it obvious that features mentioned are components of TNM staging.

That portion of the paper has now been edited as follows:

*“The cancer staging system determines the amount and spread of cancer in a patient's body and the most common practice is the use of the TNM (Tumor-Node-Metastasis) system (https://www.cancer.gov/about-cancer/diagnosis-staging/staging). T describes the size of the tumor and any spread of cancer into nearby tissue; N describes the spread of cancer to nearby lymph nodes; and M describes metastasis (spread of cancer to other parts of the body). This system was created and is updated by the American Joint Committee on Cancer (AJCC) and the International Union Against Cancer (UICC). The TNM staging system is used to describe most types of cancer. Higher performance measure and improved accuracy has been shown using a deep neural network to extract patterns from multimodal data sets for cancer staging compared with using the TNM signature often considered to be a subjective system (3–5).”* (page 2 line 36–45 in the unmarked document)

**Comment 3:**
Genomic Datasets preprocessing:
“Then an imputation function in python is used to fill out the missing values.” Please clarify if the missing values are filled with zero, or otherwise.

**Response 3:**
Thank you to the reviewer for their comment. The missing values are filled with zero, currently, we made it obvious in the text (page 6 line 6). The imputation operation was actually a ‘substitution’ operation, where we replaced missing values with zeros, performed after ensuring that genomics features that are used in the study must be present in 80% or more patients. This has been clarified as requested (line 113 in the unmarked document).

**Comment 4:**
H&E image dataset preprocessing
"we build a sub python function called MX to filter out patches with less than 30% cellular tumor content." Please, how does this function work to select the patches with >30% cellular content.

**Response 4:**

This is an important point raised. The procedure used in selecting patches with cellular content > 30% has been clarified by addition of the following statement:

“*The MX function select all patches with cellular content equal or greater than 30% through the thresholding method by estimating the patches background noise and the foreground tumour content: The patch images in RGB were converted to grayscale color and segmented into background and cell component regions. Patches with foreground content equal or greater than 70% are selected for training the prediction model.”* (lines 122– 126).

**Comment 5:**
Ensemble Deep Convolution Neural Network (EDCNN) Implementation:
The steps described in this section seem more like features integration than ensemble of models output. Also, this applies to the title of the manuscript.

**Response 5:**

We appreciate this comment and agree with the reviewer and now we changed the title of the manuscript to “*Deep Neural Network (DNN) integrating genomics and histopathological images for predicting stages and survival time-to-event in colon cancer*”

**Comment 6:**
Classification model:
“We test the validity of our hypothesis after reducing the dimension and consequently learn a higher-dimensional and compressed representation from the genomics and histopathological images.” Please provide more clarity about the higher-dimensional representation referred to here.

**Response 6:**

Thank you for pointing this out since we made a mistake in wording here; instead of ‘higher- dimensional representation’ it is supposed to be a ‘lower-dimensional’ since the NN fine-tuned and extractor model in this study have reduced the dimensions for both images and functional genomics data. The amended text is on Page 10, line 200.

**Comment 7:**
The described CNN architecture in this section seem more like an ANN.

**Response 7:**

Thank you for pointing this out, we realized that it sounds misleading, and we rephrased that statement as follows: “*The CNN classifier comprises of different layers including the input layer, convolutional layers, pooling layers and the output layer*” Page 10 line 202– 203. This is further expatiated under section 2.6

**Comment 8:**
Survival risk stratification with extracted features:
“As shown in Fig 3, the wide gap between the two functions is an indicator that we can confidently argue that 68% of the extracted features conveniently group the samples into low or high survival class.” Fig 3 only shows the result of 10 of 1836 features.

Is it possible to find out how many genomic, or image features are part of the 1836 for stratifying patients into risk groups?

**Response 8:**

Thank you to the reviewer for their comment. We have shown the top 10 distinguishing features in terms of survival stratification, five features from image features, five from genomics. This has been now clarified in lines 283-285:

“*The 1836 stratification features consist of 1204 and 632 of image and genomics features, respectively. We show the top 10 distinguishing features (five genomics, five image features), as described above, in Figure 6 in terms of their capability to present the survival stratification*.”

**Comment 9:**
Table 5:
Please explain more on the confusion matrix analysis. The number of samples in the table are not the same as those in tables 1 and 2.

**Response 9:**

Thank you to the reviewer for their comment. We have made a clarification that the confusion matrix is based only on the test data, which is 20% of 112,161 samples in Table 2. The title of the confusion matrix table has been updated and appended with 20% of test data information “Table 5 Confusion Matrix analysis for unimodal and multimodal features estimated on test data which is 20% of the entire samples”. (lines 243-244)

==================================================================

**Reviewer #2:**

This article has established an integrated deep neural network for the staging classification of colon cancer,stratifying samples into low or high-risk survival groups. There is unexplained diverse variation within the predefined stages of colon cancer. This variation can be observed when utilizing only features from either genomics or histopathological whole slide images as prognostic factors. The unraveling of this variation is expected to lead to enhanced staging and improved treatment outcomes.
Here are my suggestions:

**Comment 1:**

This article has a certain degree of novelty.The novelty of colon cancer group can be made by the data of mRNA, miRNA and DNA methylation make, but if the practical application of this group is further elaborated, this paper will be more perfect.

**Response 1:**

Thank you to the reviewer for the comment. We have amended the first part of the discussion section with a detailed update of the practical application of cancer grouping and added the following content (Page 17, lines 308 – 322 in the unmarked document):

*Cancer grouping has a vast practical application in early detection, targeted therapies and survival risk management. Research outcomes have shown that different molecular datasets are now available and have been studied for better comprehension and implementation of the mentioned cancer group application. Most available molecular datasets still required the development of novel computational techniques for the extraction of essential information leading to improved survival of colon cancer patients. Some of the major challenges requiring urgent computation techniques tools are highly related to understanding molecular states of the available molecular data such as the genetic mutation, microsatellite instability status, co-expression pattern, biological markers, methylation, and several other molecular states synonymous with different cancer stages or groups. Novel methods are needed for the analysis of several molecular states identified in the unimodal and multimodal datasets for effective and robust cancer stages early detection, targeted therapies, and survival risk management. Some of the benefits of cancer stage accurate prediction include the determination of the best treatment for the patient, estimating the chances of the cancer returning or spreading after initial treatment, determining the chances of patient recovery and the best clinical trial options. Also, cancer staging helps in verifying how new treatments work among large groups of patients with the same diagnosis.*

**Comment 2:**
If there is more clear introduction on how the randomness of the data is assured,the article will be better.

**Response 2:**

Thank you to the reviewer for their comment. To emphasize randomness and explain this better, we added a statement as follows (lines 185 – 187 in the unmarked document):

“During training Stratified 5-fold sampling and batching procedure are adopted to ensure randomness and preserve the stage frequency across each training and validation session.”

**Comment 3:**
The model established in this article was applied data from open databases without external data validation. It is desirable that more data are used to verify the accuracy of the model in further research,the application scope of this model will be wider.

**Response 3:**

This is a valid point raised for ensuring a better predictive model. This has already been stated in the concluding section of this paper as a notable limitation and as among the areas to be considered in future study as the reviewer rightly suggested.

==================================================================

**Reviewer #3:**

The authors have presented an important study integrating genomics and histopathological images for predicting stages and survival time-to-event in colon cancer. The manuscript is well written. I have the following comments:

**Comment 1:**
The methodology section needs to be put in small headings to allow a proper understanding of the methodology. This will also allow for the reproducibility of your approach.

**Response 1:**

We thank the reviewer for pointing out this pertinent suggestion, The entire manuscript sections have now been numbered accordingly. The section 2 of the manuscript contains the different steps of the methodology adopted e.g. regarding features extraction, integration of data, and model training and validation. Assigning numbers to the sections now provides a better understanding about the subsections, hence we appreciate reviewer’s comment.

**Comment 2:**
The authors mentioned that they have used 5 different sets of data from the Cancer Genome Atlas. These sets should be mentioned. Was the data from the same source? I mean if the data is from XXX Centre, does it contain all the datatypes (Clinical, DNA methylation, miRNA, mRNA, Hematoxylin, and Eosin (H&E) stained histopathological images). Hence, the specific source from TCGA should be mentioned since it is a database with numerous data.

**Response 2:**

Thank you to the reviewer for their comment. Table 1 has been updated with the assayPlatform of the genomics data. The link to dataset (TCGA-COAD) retrieved from the cancer genome atlas is provided in the manuscript (<https://portal.gdc.cancer.gov/>). The source as specified in TCGA-Assembler 2 function are: Clinical – BiospecimenClinicalData, mRNA – gene.normalized_RNAseq, miRNA – mir_Hiseq.hg19.mirbase20, DNA methylation – methylation_45, all of the datasets are from the Colon Adenocarcinoma (COAD) cancer type.

Table 1. Sample and Feature size of genomics dataset before and after preprocessing

| Biological data/assayPlatform | Before Preprocessing | | After Preprocessing | |
| --- | --- | --- | --- | --- |
|  | No. of Samples | No. of Features | No. of Overlapping Samples | No. of Features |
| Clinical/ BiospecimenClinicalData | 448 | 80 | 255 | 80 |
| mRNA/ (gene.normalized_RNAseq) | 328 | 20,502 | 255 | 16,377 |
| miRNA /(mir_Hiseq.hg19.mirbase20) | 261 | 1,870 | 255 | 420 |
| DNA methylation /(methylation_450) | 353 | 20,759 | 255 | 20,129 |

**Comment 3:**
The ethical permission statement should be mentioned.

**Response 3:**

The Ethical statement and data availability has been included as follows (lines 367-374 in the unmarked document):

***Ethical Statement:*** *All the data employed in this study is secondary data; no new data has been generated while undertaking this study.*

***Data Availability:*** *All datasets are fully available on publicly available secondary data repositories without restriction as described in the ‘materials and methodology’ section. Functional genomics datasets were downloaded from The Cancer Genome Atlas (TCGA-COAD cohort, available on* [*https://portal.gdc.cancer.gov/*](https://portal.gdc.cancer.gov/)*) and the histopathological images were downloaded from* [*https://zenodo.org/records/2530835#.YzbcnuzMLpC*](https://zenodo.org/records/2530835#.YzbcnuzMLpC)*.*

**Comment 4:**
The genomics resulted in 448 samples of clinical records with 80 features. What was the source of this data?

**Response 4:**

The source of the data is TCGA-COAD (https://portal.gdc.cancer.gov/).The source as specified in TCGA-Assembler 2 function from which the genomics data are downloaded are: Clinical – BiospecimenClinicalData, mRNA – gene.normalized_RNAseq, miRNA – mir_Hiseq.hg19.mirbase20, DNA methylation – methylation_45, all the from the COAD cancer type. Source information is updated in Table 1 (line 99 in the unmarked document).

**Comment 5:**
Also, 328 samples of mRNA expression with 20,502 features, 261 samples of miRNA expression with 1,870 features, and 353 samples of DNA methylation with 20,759 features. What was the specific source?

Response 5:

Thank you to the reviewer for their comment. The source of the data is TCGA-COAD (https://portal.gdc.cancer.gov/) The source as specified in TCGA-Assembler 2 function from which the genomics data are downloaded are: Clinical – BiospecimenClinicalData, mRNA – gene.normalized_RNAseq, miRNA – mir_Hiseq.hg19.mirbase20, DNA methylation – methylation_45, all the from the COAD cancer type. Source updated in Table1

**Comment 6:**
Regarding this statement, “We proceeded to get the Hematoxylin and Eosin (H&E) stained histopathological images for the 255 samples that have data in the genomics but got only 177 equivalent samples as in the genomics with images datasets”. Does it mean that all 177 samples were considered for clinical, DNA methylation, miRNA, mRNA, Hematoxylin, and Eosin (H&E) stained histopathological images?

**Response 6**

Yes, the research was designed for patients with datasets available in the miRNA, mRNA, DNA methylation and Histopathological images. Hence, only 177 samples satisfy this condition are considered for the four datasets. A total of 112,161 tiles were extracted from the 177 whole slide images.

**Comment 7:**
Does it mean that the deep learning model development was based on data from Table 2?

**Response 7:**

Yes, the deep learning model development was based on the data from Table 2. With 80% of the data used for training/validation and 20% of the data used for testing the model (as described in section 2.7)

**Comment 8:**
This is unclear: (i) First, a biological feature (in any of methylation or mRNA or miRNA data) is removed, if more than 20% of the patients have a 0 value for it.

**Response 8:**

This means that any genomics features that is used in the study must be present in 80% or more patients to ensure we are keeping features with a high variation ratio, reduce noise, avoid spurious association and reduce the risk of overfitting. Given that each of the genomics comprises of matrix with rows representing the patients and the columns representing biological features. The requirement is that for any of the features to use in the study – it must not have zero values in more than 20% of the patients.

**Comment 9:**
“Then an imputation function in python is used to fill out the missing values.” What imputation function was used? How many of the 177 were based on imputed data? How effective is the imputation approach. Perhaps, it may be better to remove the rows that contain missing values.

**Response 9:**

Thank you to the reviewer for raising this point. Actually, the procedure undertaken was a ‘substitution’ rather than an ‘imputation’; we now properly updated this definition (lines 118-119 in the unmarked document). The ‘substitution’ was used for filling missing values with zero values. The substitution was applied after satisfying the requirement that 80% or more features must have value in each patient, and 80% or more patients must have values in each feature. The focus of the imputation is on the features due to high dimensionality. The function for the removal of samples returned 0 (zero) hence, none of the samples has more than 20% zero values in all the features, but feature-wise 1450 features was removed from miRNA, 4125 features were removed from mRNA, and 630 features were removed from DNA methylation. We believe that removing all the rows with missing values (including the rows that have non-zero values for 80% of the participants) would be too stringent and lead to losing information.

**Comment 10:**
We input 36,926 merged features from miRNA, mRNA and DNA Methylation into autoencoder (AE) neural network design specifically to encode its input. How does the author arrive at 36,926 features?

**Response 10:**

We appreciate the reviewer for the important observation. The number of features in mRNA is 16,377, miRNA is 420 and DNA methylation is 20,129 summing up the features gives 36,926 features. This description has been included the manuscript and made clear for readers (on page 7, lines 138 – 139)

**Comment 11:**
Were the 652 extracted features combined with 2,048 salient features from deep learning extraction?

**Response 11:**

We appreciate the reviewer for the important point raised. Yes, the 652 features extracted from genomics were combined with 2048 extracted features from image tiles to get 2700 fused features. This description has been updated in the manuscript and made clear for readers. (on page 8, lines 165-166)

**Comment 12:**
The manuscript can also benefit from professional English Language proofreading.

**Response 12:**

Thank you for this suggestion: The manuscript has now been edited with Grammarly professional English Language proofreading.

**Comment 13:**
The programming code for these analyses should be inserted as an Appendix/Supplementary for reproducibility.

**Response 13:**

The programming code is available on the github link: <https://github.com/Ogundipe-L/EDCNN> for implementation.
