# Supplementary material for: Deep neural networks integrating genomics and histopathological images for predicting stages and survival time-to-event in colon cancer
Source: PLoS One. 2024 Sep 3;19(9):e0305268. doi: 10.1371/journal.pone.0305268 (PMC11371203; doi:10.1371/journal.pone.0305268)
Supplement: Attachment — Submitted filename: Response to Reviewer comments PONE-D-23-30061R1.docx [file pone.0305268.s003.docx]

Dear Editor,

We thank the reviewers for their constructive and thoughtful comments and suggestions on our manuscript originally entitled “Ensemble Deep Neural Networks (EDCNN) integrating genomics and histopathological images for predicting stages and survival time-to-event in colon cancer” and amended to “Deep Neural Network (DNN) integrating genomics and histopathological images for predicting stages and survival time-to-event in colon cancer” upon a comment from one of the reviewers (Manuscript Number: PONE-D-23-30061R1). We have revised the manuscript to address each of the comments. Below are our point-to-point responses to the reviewers’ comments.

The modifications in the manuscript are highlighted within the “Revised-PONE-D-23-30061R1-with-track.docx” document. We also provided an unmarked version of the revised document (Revised-PONE-D-23-30061R1-unmarked.docx) upon your request.

Sincerely,

Dr Zeyneb Kurt,

Lecturer in Data Science,

The University of Sheffield, The Information School

**Reviewer #1:**

**Comment1:**

Thank you very much for your response to the previous comments, and for the changes made.

As regards the the confusion matrix on table 5, the table shows a total of 112,161 samples that were predicted by the models. This applies to each of genomic, images, and the integrated data. However, only image samples contain a total of 112,161 tiles.

**Response 1:**

Thank you to the reviewer for their valuable comment. We have added a description of our mapping method and the projection of each dataset modality into 112,161 samples under Section 2.5 as highlighted under response 2 below. Also, according to the caption of the confusion matrix shown in Table 5, the estimations were based on 20% of the 112,161 entire dataset which is approximately equivalent to 22,432 size of testing datasets. At the patient’s level, this represents 35 (20%) samples of the 177 patients. Hence, Table 5's confusion matrix was calculated using a testing dataset of 35 patients projected into 22,432 samples.

**Comment2:**

On the patient level, the genomic data contain 177 samples, and same for image data too. If this is correct, I would expect at least the the number of genomic, and integrated samples to be 177. Please, can you clarify more on these? Were the image tiles eventually aggregated to patient level? what methods was used for the aggregation?

**Response 2:**

Thank you to the reviewer for their valuable comment. We have added a description of our mapping method and result analysis under Section 2.5 as follows (Page 8):

“*The feature extraction process from image tiles and the genomics datasets concluded with the dimensions of 112,161(samples)x2048(dimensions) and 177(samples)x652(dimensions), respectively. We used a many-to-one technique to concatenate vectors from the images and genomics datasets, requiring that concatenated features from both sides (image and genomics) have the same patient identification code. The concatenation procedure generates a fused dataset of size 112,161(samples)x2700(dimensions).*

*To guarantee that an equal number of rows from each of the three modality options (i.e. (i) images only, (ii) genomics only, (iii) fused image and genomics) are utilized for training the prediction models, the complete fused dataset is labeled as the integrated dataset with dimensions 112,161x2700.*

*The datasets with 112,161 rows in each modality were divided into 89,729 (80%) for the training and 22,432 (20%) for model testing. At the patient’s level, this represents 142 (80%) samples for training and 35 (20%) samples for testing.*

*The analysis and result of the study were based on the testing portion of the datasets, equivalent to 22,432 (20%) in each modality.*”

For further clarification: we have not aggregated the data or results in patient level; instead, we presented the results based on multiplied data due to the concatenation operation.
